# The Influence Mechanism of Dissolved Organic Matter on the Photocatalytic Oxidation of Pharmaceuticals and Personal Care Products

**DOI:** 10.3390/molecules30112266

**Published:** 2025-05-22

**Authors:** Jie Wang, Minyi Zhu, Anli Sun, Rongfang Yuan, Huilun Chen, Beihai Zhou

**Affiliations:** Beijing Key Laboratory of Resource-Oriented Treatment of Industrial Pollutants, School of Energy and Environmental Engineering, University of Science and Technology Beijing, Beijing 100083, China

**Keywords:** dissolved organic matter, photocatalytic oxidation, pharmaceuticals and personal care products, influence mechanism

## Abstract

With the worsening global water pollution crisis, pharmaceuticals and personal care products (PPCPs) have been increasingly detected in aquatic environments. The effective removal of PPCPs remains challenging for conventional water treatment technologies, whereas photocatalytic technology has shown distinct promise. Dissolved organic matter (DOM), a ubiquitous component of aquatic ecosystems, exerts multifaceted effects on the photocatalytic oxidation of PPCPs. In this article, the influence of DOM on the performance of various photocatalysts in PPCP removal is systematically summarized and analyzed. This review highlights DOM’s role in altering the migration and transformation of PPCPs via processes including adsorption and complexation. The adsorption of PPCPs on photocatalysts is achieved by competitive adsorption or by providing more adsorption sites. DOM modifies the structural properties of photocatalysts through mechanisms such as ligand exchange, intermolecular forces, electrostatic forces, and hydrophobic interactions. DOM inhibits the formation of active species via light attenuation and shielding effects while simultaneously enhancing their generation through photosensitization and electron transfer facilitation. In this review, the interaction mechanism among DOM, PPCPs, and photocatalysts within the PPCP photocatalytic oxidation system is expounded on. These findings provide novel insights into optimizing photocatalytic reaction conditions and enhancing treatment efficiency, while providing a theoretical foundation for advancing efficient, eco-friendly PPCPs remediation technologies.

## 1. Introduction

Globally, water pollution has emerged as a critical challenge threatening human health and ecological balance. Water pollution not only compromises the security of water for daily use but also profoundly impacts aquatic ecosystems and biodiversity. Pharmaceuticals and personal care products (PPCPs) have emerged as a globally prevalent class of contaminants detected in aquatic systems. In recent years, PPCPs have been frequently detected at elevated concentrations in aquatic environments, posing significant ecological risks to aquatic organisms and human health [1]. For instance, in rural Czech wastewater systems, the median mass concentrations of caffeine, ibuprofen, and paracetamol exceeded 10,000 ng·L^−1^, with diclofenac, atenolol, and metoprolol concentrations surpassing 1000 ng·L^−1^ [2]. Similarly, elevated concentrations of N,N-Diethyl-3-methyl benzoyl amide (DEET), sulfamethoxazole, and tramadol in Jakarta Port, Indonesia, were also at a relatively high level [3]. In China’s Yangtze River, DEET concentrations in surface water have reached 166 ng·L^−1^ [4], while oxytetracycline levels in the Pearl River Basin peaked at 2030 ng·L^−1^ [5]. The sources of DEET in rivers mainly include the discharge of domestic sewage and industrial wastewater, and the runoff and infiltration of agricultural, forestry, and horticultural wastewater. Oxytetracycline belongs to the tetracycline class of antibiotics, which can be discharged into rivers through medical wastewater, agricultural runoff, and pharmaceutical wastewater. In Tangxun Lake and East Lake, bisphenol A and estrone were identified as high-risk contaminants, whereas triclosan and estriol exhibited moderate risks in certain areas [6].

Given the extensive pollution problems caused by PPCPs, traditional water treatment technologies struggle to completely remove these complex organic pollutants. As an advanced oxidation technology, solar-driven photocatalysis demonstrates unique advantages in the treatment of PPCPs due to its low energy consumption, environmental friendliness [7], and high yield of reactive oxygen species (ROS) [8]. Semiconductor-based photocatalysts leverage their optoelectronic properties to enable energy-efficient pollution control. For instance, S-C_3_N_5_ achieved a sulfamethoxazole degradation efficiency exceeding 90% after 30 min of visible light exposure [9], while Bi/W_18_O_49_ demonstrated removal rates of 93%, 87%, and 62% for trimethoprim, acetaminophen, and tetracycline, respectively, under 610 nm red light irradiation [10]. Similarly, TiO_2_ nano-dispersions degraded tetracycline by over 90% within minutes under both visible and ultraviolet light [11], demonstrating the favorable effect of photocatalytic technology in degrading PPCPs.

Dissolved organic matter (DOM), ubiquitous in natural aquatic environments, comprises organic substances that dissolve in water and can pass through a 0.45 μm filtration membrane [12]. The primary components of DOM in surface water include humic acids, polysaccharides, and proteins [13], along with small molecular organic acids and other components [12]. In practical research, DOM concentrations are typically quantified using dissolved organic carbon (DOC). In surface water and groundwater, DOM concentrations typically range from 1 to 10 mg·L^−1^. In most treated wastewater, concentrations generally range between 5 and 30 mg·L^−1^ [14], and the overall concentration range in the water environment is 0.5–100 mg·L^−1^ [12]. Given that DOM concentrations far exceed those of PPCPs in aquatic environments, DOM inevitably mediates the interactions between PPCPs and particulate matter or sediments.

Owing to their abundant functional groups and dual ionization structures, PPCPs exhibit a strong tendency to bind with DOM in aquatic environments, significantly affecting their migration, transformation, and removal during the wastewater treatment process [15]. This interaction not only alters the physicochemical forms and stability of PPCPs but also exerts a multifaceted influence on their degradation efficiency in photocatalytic systems. As a natural organic component in the water, DOM possesses diverse functional groups and reactive sites that promote strong interactions with PPCP molecules, resulting in stable complexes.

In addition to the interaction with PPCPs, some components in DOM, especially organic compounds rich in functional groups such as carboxyl and hydroxyl groups, can bind to photocatalysts’ surfaces via electrostatic interactions, hydrogen bonds, or coordination bonds. Such binding can occupy or block active sites on photocatalyst surfaces, limiting PPCPs’ access to reactive sites and thereby diminishing photocatalytic efficiency. Furthermore, DOM adsorption on photocatalyst surfaces can alter surface charge distribution and energy band structures, impacting light absorption and photogenerated charge carrier separation efficiency, ultimately modulating the kinetics of photocatalytic reactions.

Under illumination, certain DOM fractions exhibit photosensitization, generating reactive species such as excited triplet states (^3^DOM*), hydroxyl radicals, and superoxide radicals, which promote the degradation of PPCPs. Conversely, studies have demonstrated that DOM can inhibit the photodegradation of organic substances through light screening, scavenging of reactive species, and reaction intermediates [16,17]. For instance, DOM acting as a quencher of photoexcited molecules has been shown to reduce the degradation rates of PPCPs (e.g., cimetidine, carbamazepine, and propranolol) by photocatalysts like TiO_2_ and ZnO [18,19]. DOM has been observed to inhibit PPCPs’ degradation under visible or UV light irradiation through light shielding and light attenuation mechanisms. For example, DOM reduces the degradation of metronidazole by UV/TiO_2_ and the photocatalytic degradation process of organic substances such as propranolol, diclofenac, and carbamazepine by TiO_2_ [20,21].

This paper summarizes the influence of DOM on the photocatalytic oxidation of PPCPs. The impacts of DOM on the migration and transformation of PPCPs, the surface and interface structures of photocatalytic materials, and the reactive species in the reaction system are reviewed. The paper elucidated the mechanism of how DOM influences the photocatalytic oxidation of PPCPs and identified current challenges that require addressing.

## 2. Effect of DOM on the Photocatalytic Oxidation of PPCPs

**Figure 1:**
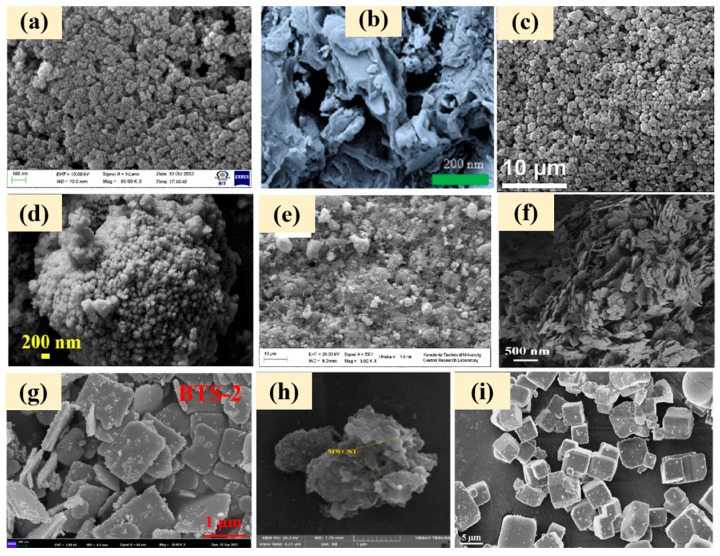
SEM of different types of photocatalytic materials. (**a**) TiO_2_ NPs [22]; (**b**) g-C_3_N_4_ [23]; (**c**) CdS [24]; (**d**) BiVO_4_ [25]; (**e**) ZnO/g-C_3_N_4_ [26]; (**f**) Bi/Bi_2_WO_6_ [27]; (**g**) BiOBr/black-TiO_2_/tourmaline composites [28]; (**h**) TiO_2_/MWCNT/PANI [29]; (**i**) SnO_2_-x/CD-MOF S-Scheme [30].

Photocatalytic materials are a category of catalytic materials that can be excited to generate electron–hole pairs under light illumination and achieve environmental purification through oxidation-reduction reactions. Based on their composition and structural characteristics, these materials can be classified into metal oxide-based, carbon-based, metal sulfide-based, bismuth-based, and composite photocatalytic materials and newly emerged nanocatalytic materials. The structural characteristics of several common photocatalytic materials are shown in Figure 1.

The effect of DOM on the degradation of PPCPs by photocatalytic materials is complex and diverse. Table 1 lists the degrees of influence of DOM on the degradation of PPCPs by different types of photocatalytic materials.

### 2.1. Metal Oxide-Based Photocatalysts

In recent years, metal oxide-based photocatalysts have garnered significant attention from researchers in the field of organic compound degradation. This is attributed to their exceptional light absorption capabilities under both visible and ultraviolet light irradiation and their favorable safety and stability properties [70]. However, the photocatalytic performance of metal oxide-based photocatalysts is often limited by factors such as the rapid recombination of electron–hole pairs generated by light, limited light absorption in the visible spectrum, and low surface area. To enhance the performance of metal oxide-based photocatalysts, various modification methods can be adopted. At present, the most common modification methods include doping metals [71] or non-metals [72], sensitizing with dyes [73] and quantum dots [74], constructing heterojunctions [75], and loading co-catalysts (noble [76] or non-noble metals [77]), etc. These modification methods can significantly improve the performance of metal oxide-based photocatalysts.

#### 2.1.1. TiO_2_

TiO_2_ is widely recognized as one of the most effective semiconductor photocatalysts, offering advantages such as high chemical stability, strong photocatalytic activity, non-toxicity, and low cost. DOM has a significant influence on the catalytic effect of TiO_2_-based photocatalysts. For instance, the presence of 10 mg·L^−1^ humic acid (HA) can decrease the reaction rate constant (k) for the degradation of bupropion (BUP) by TiO_2_ from 7.5 × 10^−2^ min^−1^ to 1.5 × 10^−2^ min^−1^, resulting in an inhibitory effect of nearly 80% [31]. This phenomenon occurs because DOM can interfere with the decomposition of TiO_2_ to target pollutants through occupying surface sites, scavenging free radicals, holes, and absorbing ultraviolet light [78]. However, DOM exhibits minimal impact on the catalytic effect of TiO_2_ nanotube arrays (TiO_2_ TNAs). This resistance is attributed to their larger specific surface area, which provides more active sites.

The influence of DOM on the degradation of organic matter by TiO_2_ is strongly related to the crystal form of TiO_2_ [32]. There are seven crystal forms of TiO_2_, but only rutile, anatase, and brookite exist in nature [79]. The most commonly used crystal form of TiO_2_ is anatase. Compared to rutile, which has four Ti–O bonds, anatase exhibits more pronounced octahedral distortions. Anatase, with two Ti–O bonds, is more prone to defect formation, thus generating more electron holes. Additionally, anatase possesses a larger surface area [80], which enhances the efficiency of the photocatalytic reaction [70]. Lee et al. [32] investigated the influence of HA on anatase and rutile. During the degradation of 4-chlorophenol (4-CP), anatase generates ·OH through the O_2_ reduction pathway, which is inhibited by the competitive adsorption of HA and H_2_O_2_. Rutile primarily generates ·OH through the oxidation of H_2_O. When the HA concentration is below 20 mg·L^−1^, HA facilitates greater electron participation in the reduction of O_2_, enhancing the yield of ·O_2_^−^ and promoting the degradation of 4-CP.

#### 2.1.2. ZnO

ZnO, a nano-semiconductor material, is extensively utilized in solar cells, lithium-ion batteries, and photocatalysis [81]. It has been widely employed for degrading environmental pollutants in air and water, and converting selective organic pollutants into non-toxic small molecules or even into CO_2_ or H_2_O [82]. ZnO is a wide-bandgap semiconductor that can only absorb ultraviolet light to generate photoelectrons and holes [81]. As a member of the II-VI group, ZnO has an optical bandgap of 3.26 eV. Owing to its tunable band structure, it has emerged as a promising alternative and photocatalyst [83].

During the process of ZnO photocatalytic degradation of tetracycline (TC) by ZnO under visible light, the addition of HA resulted in a 19% reduction in TC degradation efficiency [34]. The study by Li et al. [84] demonstrated that a specific concentration of HA (5 mg·L^−1^) significantly inhibits ·OH generation by ZnO under UV irradiation. Complexation between Zn^2+^ released into the solution and HA deactivates the triplet state of HA, thus hindering the degradation of organic pollutants by ZnO.

Compared with ZnO, ZnO nanomaterials are more significantly affected by DOM. The introduction of DOM caused the removal rate of methylene blue (MB) by ZnO nanoparticles (ZnO NPs) to decrease by 79.4%, and it also caused the removal rate of cefalexin by ZnO nanowires to drop sharply from 100% to 40.9% [36]. This strong inhibitory effect is attributed not only to the quenching of active free radicals by DOM, the attenuation and consumption of light, and the occupation of the active sites of ZnO nanomaterials [82], but also the ability of HA to promote the dissolution of ZnO nanoparticles, with solubility increasing as the HA concentration rises [85]. Therefore, the catalytic degradation efficiency of ZnO nanomaterials is significantly impacted.

#### 2.1.3. WO_3_

As an n-type semiconductor, WO_3_ is extensively employed as a photocatalyst for pollutant degradation owing to its high chemical stability, electron mobility [59], relatively narrow bandgap (2.6–2.8 eV [86]), and strong oxidation-reduction potential [87]. However, WO_3_ is characterized by a small specific surface area [88], a high carrier recombination rate, and a low quantum yield. Therefore, coupling WO_3_ with functional materials to form a heterostructure or doping and modifying are effective strategies for enhancing its photocatalytic performance [50,89].

Yazdanbakhsh et al. [37] doped Mn into WO_3_ and investigated the influence of varying HA concentrations on the degradation of diclofenac (DCF) by Mn-WO_3_. The results showed that the inhibitory effect of HA on the reaction system became increasingly pronounced as its concentration increased. Xu et al. synthesized a WO_3_/ZnIn_2_S_4_ Z-scheme heterojunction and observed that the inhibitory effect (53.9%) of the addition of HA on the degradation of tetrabromobisphenol A (TBBPA) was significantly higher than that of inorganic anions, including Cl^−^, HCO_3_^−^, and SO_4_^2−^. This phenomenon can be attributed to the scavenging effect of HA on free radicals and its light shielding effect, both of which hinder pollutant degradation [59].

### 2.2. Carbon-Based Photocatalysts

Carbon-based materials possess a large surface area, high mechanical strength, excellent electron mobility, thermal conductivity, and carrier mobility, making them essential for the removal of organic pollutants in water [90].

#### 2.2.1. Graphitic Carbon Nitride (g-C_3_N_4_)

The polymer semiconductor g-C_3_N_4_ is one of the carbon-based photocatalytic materials that have been extensively investigated in recent years. It exhibits a relatively large specific surface area, a stable energy gap (~2.7 eV), excellent electronic properties, and rich functional groups with abundant surface defects. Therefore, it has been widely used in the removal of environmental pollutants [39].

The influence of DOM on the degradation efficiency of g-C_3_N_4_-based photocatalytic materials is also dualistic. Due to the reaction between DOM and photogenerated holes (h^+^) or the competitive adsorption with PPCPs, the addition of DOM can inhibit the degradation of the target pollutants. For instance, the addition of HA resulted in an 18% reduction in the degradation rate of phenanthrene (PHE) by g-C_3_N_4_ [39]. Conversely, Meng et al. [38] found that the presence of HA has an obvious promoting effect on the degradation of carbamazepine (CBZ), with the promoting effect being positively correlated with HA concentration. This phenomenon may be attributed to the coadsorption or accumulation of HA and CBZ on the catalyst, promoting its degradation.

#### 2.2.2. Graphene

Graphene, a honeycomb structure of six-membered rings formed by carbon atoms through sp^2^ hybrid orbitals [91], exhibits exceptional electrical conductivity, thermal conductivity, and a unique quantum tunneling effect [92]. Consequently, the integration of photocatalysts with graphene can further promote the migration of photogenerated electrons and significantly improve the performance of photocatalysts [93].

Zou et al. [40] investigated the influence of fulvic acid (FA) on the degradation of acetaminophen (APAP) by graphene oxide (GO) and found it to be significant. The addition of 20 mg·L^−1^ FA reduced the reaction rate constant from 0.4547 min^−1^ to 0.0689 min^−1^. This reduction is attributed to the consumption of photogenerated holes and radicals during the photodegradation of FA and the competitive adsorption and oxidation of FA and APAP on the surface of reduced graphene oxide (RGO), which collectively inhibit the reaction [94]. However, Chen et al. [52] demonstrated that a low concentration of FA can generate reactive species, such as ^1^O_2_ and ^3^FA*, under visible light irradiation, thereby promoting the degradation of DCF. In contrast, a high concentration of FA produced a light shielding effect, which inhibited DCF degradation.

#### 2.2.3. Other Carbon-Based Photocatalysts

Fullerenes and carbon nanotubes (CNTs) are widely utilized carbon-based photocatalysts. A fullerene is a hollow molecule composed exclusively of carbon atoms. Based on the total carbon atom count, fullerenes are classified as C_20_ [95], C_60_ [96], C_70_ [97], etc. They exhibit strong UV absorption alongside relatively weak visible light absorption [97]. C_60_ has exceptional redox performance due to its high electronegativity and strong antioxidant capacity, enabling efficient electron acceptance [98]. In addition, fullerenes are often used in combination with various wide bandgap semiconductor photocatalysts to achieve the purpose of efficient degradation of pollutants, such as TiO_2_ [99] and ZnO [100].

CNTs are tubular nanostructures formed by rolling single- or multi-layer graphene sheets. These carbon allotropes exhibit high aspect ratios, unique electronic properties, and surface functionalization potential. Based on layer configuration, they are categorized into single-walled carbon nanotubes (SWCNTs) [101] and multi-walled carbon nanotubes (MWCNTs) [102]. SWCNTs can exhibit metallic or semiconductor properties, while MWCNTs have a more complex band structure due to their multilayer structure.

Li et al. [103] employed electron spin resonance spectroscopy (EPR) to investigate the effects of HA under light on the induction of singlet oxygen and hydroxyl radicals by typical carbon nanomaterials. In the co-presence with HA, both fullerenes and CNTs significantly induce the production of ·O_2_^−^, and fullerenes can also photosensitively generate ·OH. The ability to cooperatively produce ^1^O_2_ is as follows: SWCNTs > fullerenes > MWCNTs. Another report from Zhang et al. [104] reported that HA adsorption on the C_60_ surface promotes the dispersion through electrostatic repulsion and steric stabilization. This uniform dispersion enhances catalyst–pollutant contact, thereby accelerating pollutant degradation. These findings suggested that the presence of DOM may promote the oxidation effect of such carbon-based photocatalysts on PPCPs, though the effect varies among composite systems.

### 2.3. Metal Sulfide-Based Photocatalysts

Compared to metal oxides, metal sulfides possess fewer valence bands in the sp^3^ orbitals, resulting in a broader light response range and a higher carrier concentration [105].

Cadmium sulfide (CdS), a metal sulfide semiconductor material, exhibits a strong photoelectric effect. Due to its relatively narrow bandgap, CdS can absorb a wider range of the visible light spectrum from solar radiation, enabling its widespread application in optoelectronic devices and related fields. The bandgap of CdS is 2.4 eV [106]. When the CdS semiconductor absorbs the electromagnetic radiation from solar radiation, an excited state is formed, causing valence band electrons to transition to the conduction band. This process generates highly redox-active electron–hole pairs, which facilitate the degradation of organic pollutants in wastewater.

The influence of DOM on metal sulfide-based photocatalytic materials also has dual characteristics. Jiang et al. [41] loaded CdS onto biochar to synthesize CdS@BC for thiamethoxam (THM) degradation. Their study revealed slight HA-induced inhibition of THM degradation. Conversely, Zhang et al. [63] reported that HA at concentrations of 0–10 mg·L^−1^ has a slight promoting effect on the degradation of tetracycline (TC) by IS-Ni_2_P/CdS@g-C_3_N_4_. Similarly, Du et al. [49] demonstrated that HA enhances the degradation of 17β-estradiol (17β-E2) by Er^3+^-CdS/MoS_2_, which is attributed to the photosensitization effect of DOM.

### 2.4. Bismuth-Based Photocatalysts

Among photocatalytic materials, bismuth-based semiconductors have garnered significant attention due to their excellent biocompatibility, chemical stability, and relatively narrow bandgap (most < 3.0 eV) [107]. The valence bands of compounds containing Bi^3+^ are formed by the hybridization of O 2p and Bi 6s^2^ orbitals, leading to an elevation of the valence bands [107]. Bismuth-based photocatalysts primarily include bismuth oxide, bismuth oxyhalide, bimetallic oxides, etc.

For most bismuth-based photocatalytic materials, DOM shows an obvious inhibitory effect, with the inhibition rate ranging from 5% to 45%. For example, the addition of DOM resulted in a 30% reduction in indomethacin (IDM) during SrBiOI photocatalysis [42]. This phenomenon may be attributed to the light shielding effect of DOM and the consumption of active species, which significantly decrease the degradation efficiency of IDM. However, Fan et al. [44] investigated the influence of FA and HA on the photocatalytic degradation of naproxen (NPX) by bismuth titanate nanobulk (Bi-TNB) and found that low-concentration (5 mg·L^−1^) HA can double the degradation rate of NPX, whereas FA consistently exhibits an inhibitory effect. Liu et al. [43] synthesized molecularly imprinted BiOCl (MI-BiOCl) and observed that the addition of HA did not significantly inhibit the degradation of venlafaxine (VEN). This suggests that MI-BiOCl with imprinting sites can selectively adsorb VEN without adsorbing HA, thus effectively using radicals to degrade VEN.

### 2.5. Composite Photocatalysts

Composite photocatalysts include semiconductor composites [108], carbon material composites [109], metal composites [110], organic polymer composites [111], sensitizer composites [112], etc. Based on the composite method, they can be divided into binary [113] and ternary [114] photocatalysts, etc.

The influence of DOM on the photocatalytic effect of composite materials is predominantly inhibitory, although it can exhibit a promoting effect in certain cases. For example, most carbon-based composite materials under visible light irradiation will be inhibited by DOM. However, under blue light irradiation, low-concentration (≤10 mg·L^−1^) FA can promote the degradation of DCF by rGO/TiO_2_. This phenomenon is speculated to be related to the specific excitation effect of blue light on FA molecules. Additionally, during the photocatalytic degradation of TC by IS-Ni_2_P/CdS/CN and MIL-88B(Fe)/ZnTi-LDH, HA also plays a promoting role in degradation, which may be due to the photosensitization effect of HA or the indirect generation of ·OH accelerating the degradation of TC.

### 2.6. Novel Photocatalysts

Currently, some novel nanomaterials show great potential in the field of photocatalysis, primarily including metal–organic frameworks (MOFs) [115], covalent organic frameworks (COFs) [115], MXene [116], etc. These materials are characterized by their large specific surface area, easy recovery, and strong controllability.

For such catalysts, DOM still mainly exhibits an inhibitory effect. For example, Xu et al. [65] investigated the influence of DOM on the photocatalytic degradation of 2,2′,4,4′-tetrahydroxybenzophenone (BP-2) by BMOF-Ti/Zr6%. They observed that the addition of HA and FA inhibited the adsorption of BP-2 on MOF, and the inhibitory effect of FA was more obvious. The same inhibitory phenomenon also appears in the photocatalytic process of PPCPs by materials such as Zr-MOFs [66] and π-COF [67]. Since such catalytic materials have a large specific surface area, they possess strong adsorption capacities. It is hypothesized that the competitive adsorption of DOM on the material surface inhibits the degradation effect of the target pollutants.

However, in the study by Zhang et al. [68], HA promoted the degradation of bisphenol A (BPA) by ZnFe_2_O_4_-seed@TpTt-COF. This is attributed to the presence of quinone functional groups in HA, which can act as active sites, and its rich functional groups can serve as electron donors and electron shuttles [117], thereby accelerating the degradation of BPA.

### 2.7. Chapter Summary

Overall, the influence of DOM on the photocatalytic oxidation of PPCPs is predominantly inhibitory. This phenomenon is particularly evident in metal oxide-based, carbon-based catalysts, and their composite materials, with the highest inhibitory effect reaching up to 84.8%. In contrast, the promoting effect of DOM is mainly observed in novel photocatalytic materials, such as COFs, and modified photocatalysts that have undergone elemental doping or surface modification. These materials typically exhibit an optimized electronic structure and enhanced light absorption capacity, which may facilitate electron transfer processes when interacting with DOM. In particular, for the same catalytic material, the effect of DOM shows an obvious dependency on the concentration. For example, low concentrations of DOM can promote carrier separation through photosensitization, thereby accelerating the degradation of pollutants. Conversely, high concentrations of DOM significantly inhibit the catalytic activity due to the light shielding effect and competitive adsorption of active sites. This concentration-dependent effect reveals the dualistic mechanism of DOM on the photocatalytic oxidation of PPCPs, that is, there are both the promoting effect mediated by electron transfer and the inhibitory effect caused by light absorption competition.

## 3. Effect of DOM on the Migration and Transformation of PPCPs

**Figure 2:**
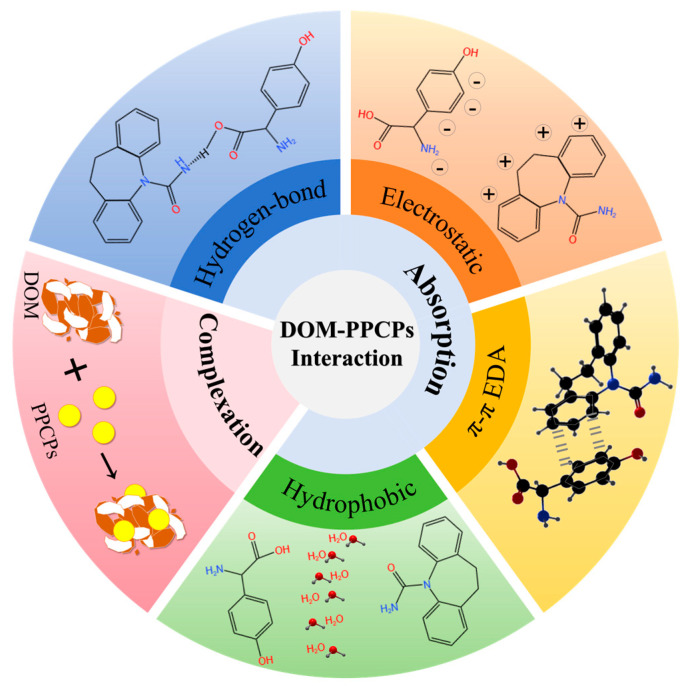
Interaction mechanism between dissolved organic matter (DOM) and pharmaceuticals and personal care products (PPCPs).

In the past few decades, people’s awareness of the ecological risks of PPCPs has been increasingly enhanced. When PPCPs are released into the aquatic environment, it is essential to understand their migration and transformation pathways. Migration, adsorption, bioabsorption, and degradation are the main behaviors of PPCPs following their introduction into the environment, and the role of DOM in these processes cannot be ignored. The interaction mechanism between DOM and PPCPs is shown in Figure 2.

### 3.1. Adsorption

During the interaction between DOM and PPCPs, the influence of DOM is particularly significant for highly polar PPCPs, such as certain antibiotics like tetracyclines and sulfonamides. The polar functional groups in these antibiotic molecules, such as the amino group and carboxyl group, facilitate interactions with the polar components of DOM, such as HA and FA. The adsorption of DOM can reduce the environmental mobility of these antibiotics, limiting their diffusion in soil and water bodies, thus potentially affecting the environmental fate and ecological risks of antibiotics. The adsorption mechanisms between DOM and PPCPs include π–π electron donor–acceptor (π–π EDA) interaction, hydrogen bonding, electrostatic interaction, hydrophobic interaction, etc.

Aolin et al. [118] showed that hydrophobic interaction plays an important role in the binding of PPCPs and DOM. Compared to FA, HA has a larger molecular weight distribution, a narrower molecular size distribution, and lower polydispersity [119], and the interaction between HA and PPCPs is more obvious.

The adsorption process between DOM and PPCP molecules typically involves multiple mechanisms acting in combination. For instance, Sun et al. [120] investigated the adsorption properties of coal humic acid (CHA) and soil humic acid (SHA) toward TC, indicating that hydrogen bonding and electrostatic interaction are key mechanisms of the interaction between TC and the functional groups in HA. Similarly, Niu et al. [121] showed that the strong hydrophobic interaction and π–π EDA between PHE and straw biochar DOM (BDOM) led to a high adsorption capacity of PHE on the BDOM400 adsorbent.

### 3.2. Complexation

DOM can form complex structures with PPCPs through chelation and ionic bonds. Yang et al. [122] pointed out that the presence of DOM reduces the overall electrochemical potential of the solution, and the complexation of DOM with triclosan (TCS) increases its molecular weight, thereby inhibiting the migration and transport of TCS across the ion exchange membrane. With rich functional groups and a large specific surface area, humus can bind to PPCP molecules. For example, PPCPs rich in amino acids can form covalent bonds with phenolic humus [123], promoting their adsorption in soil or sediment, thereby reducing their migration. Yao et al. [123] explored the influence of DOM coupling on the migration of 33 kinds of PPCPs, indicating that higher DOM abundance and aromaticity enhance the migration rate of PPCPs at the soil–water interface through co-transport and competitive adsorption. Humus-like DOM negatively impacts PPCPs, whereas proteinaceous DOM exhibits a higher affinity for PPCPs. Proteinaceous DOM contains functional groups such as the amino group and carboxyl group, which can undergo complexation reactions with PPCPs to form larger complexes, affecting the mobility of PPCPs. Studies by Huang et al. [124] and Ma et al. [125] further confirmed that DOM with a higher molecular weight has more obvious aromaticity; contains more acidic groups, fatty acids, long aliphatic side chains, and polysaccharides; and is thus more likely to chelate with PPCP molecules.

The colloidal environment formed by polysaccharides can encapsulate certain PPCPs, thereby hindering their degradation. In addition, in aquatic environments, polysaccharide-type DOM disintegrates under microbial activity, producing acidic or alkaline substances that change the environmental pH value. For example, when the decomposition of polysaccharides acidifies the water body, some alkaline PPCPs such as sulfonamide drugs may be more likely to dissolve and migrate.

## 4. Interference Mechanism of DOM on the Surface and Interface Structure of Photocatalytic Materials

### 4.1. Effect of DOM on the Adsorption of PPCPs by Photocatalytic Materials

DOM is commonly present in aquatic environments, and its competitive adsorption with PPCPs or photocatalytic materials is a key factor influencing the adsorption of PPCPs on photocatalysts. DOM can undergo competitive adsorption on the catalysts’ surfaces through hydrophobic interactions, π–π EDA interactions, hydrogen bonding, and electrostatic interactions, thereby leading to an enhancement or weakening of the adsorption of PPCPs [126].

The micropore filling effect explains why adsorbents can efficiently adsorb low-molecular-weight compounds. Compared to macromolecular natural DOM, DOM with a smaller molecular size can compete for the adsorption sites within micropores [127], thus affecting the adsorption of PPCPs on the catalysts’ surfaces. Furthermore, more hydrophobic DOM, such as HA, can compete for adsorption sites on the catalysts’ surfaces through hydrophobic interactions [128].

Studies have demonstrated that the competitive adsorption of DOM on catalysts significantly reduces the degradation rates of PPCPs, such as fibrates, carbamazepine, and sulfamethoxazole, by photocatalysts like TiO_2_ and MCNT-TiO_2_ [21,129]. Liu et al. [130] found that the presence of HA reduces the adsorption of highly organic compounds, such as ketoprofen, carbamazepine, and BPA, on catalysts. Similarly, Liao et al. [131] pointed out that HA competes with norfloxacin (NOR) and sulfamethoxazole (SMX) for the polar groups and hydrophobic sites on the adsorbent through hydrogen bonding and π–π interactions.

Under different pH conditions, the extent of DOM influence on the adsorption of organic pollutants by catalysts differs significantly. The study by Ye et al. [132] showed that acidic conditions are favorable for the adsorption of DOM [133,134], while alkaline conditions reduce the competitive adsorption of DOM, thereby promoting the degradation of pollutants.

Conversely, DOM complexed with catalysts can also improve the adsorption of PPCPs on the catalysts’ surfaces by providing additional adsorption sites, such as oxygen-containing functional groups and aromatic rings, or by reducing the electrostatic repulsion between PPCPs and catalysts [135]. For example, DOM enhances the adsorption of TC on functionalized oxidized graphene nanoparticles [136] and the adsorption of propranolol (PRO) on MWCNTs [137]. Lin et al. [138] further showed that HA bound to the surface of MWCNTs introduces oxygen-containing functional groups and negative charges, thus significantly increasing the adsorption capacity of MWCNTs for Pb^2+^.

### 4.2. Effect of DOM on the Apparent Structure of Photocatalysts

**Figure 3:**
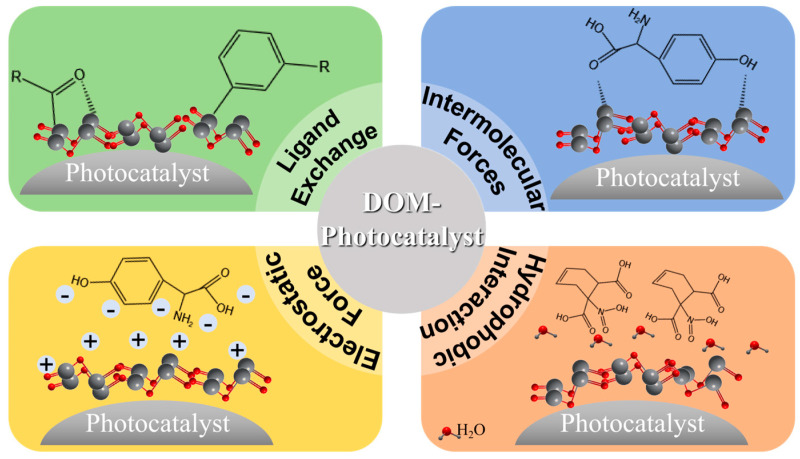
Effect of DOM on the apparent structure of photocatalysts.

DOM contains a variety of functional groups that can interact with the surface of photocatalysts through mechanisms such as ligand exchange, intermolecular forces, electrostatic force, and hydrophobic interaction (as shown in Figure 3), thereby influencing the photochemical properties of the photocatalysts [139].

#### 4.2.1. Ligand Exchange

The main components of DOM include tyrosine, FA, HA, polysaccharides, lipids, proteins, and other organic substances. [140]. DOM is rich in various functional groups, including aldehyde, amino, carboxyl, ester, hydroxyl, ketone, and phenol groups [141]. The oxidized functional groups in DOM exhibit strong electrophilicity, making them prone to pairing with other functional groups on the surface of the photocatalytic materials (e.g., amino and hydroxyl groups) to form stable compounds. Notably, DOM contains a relatively large number of acidic functional groups, such as carboxyl groups and phenolic hydroxyl groups, which can undergo ligand exchange reactions with the functional groups on the surface of the photocatalytic materials [140]. For example, Li et al. [142] demonstrated that the surface complexation–ligand exchange reaction between the surface of HA and nano-TiO_2_ resulted in the disappearance of the HA phenolic hydroxyl groups peak at 1245 cm^−1^, indicating a strong interaction between the phenolic hydroxyl groups and the surface of nano-TiO_2_.

#### 4.2.2. Intermolecular Forces

When DOM coexists with photocatalysts, various intermolecular forces arise between them, including Coulomb force, van der Waals forces, hydrogen bonds, and covalent bonds [13].

For instance, DOM containing a high proportion of polar functional groups, such as carboxyl and hydroxyl groups, can form strong bonds with the surface of the photocatalytic material through hydrogen bonds. By analyzing Fourier transform infrared (FTIR) spectroscopy, Dong et al. [143] observed that after HA was adsorbed on Mo-Se/OHNT, the formation of hydrogen bonds caused a significant red shift in the O–H/N–H stretching vibrations and a slight blue shift in the bending vibrations. When OHNT was calcined to remove surface hydroxyl groups and the HA adsorption experiment was repeated, no hydrogen bonds were formed. This further confirmed that hydrogen bonds formed between the hydroxyl/amino of HA and the hydroxyl of Mo-Se/OHNT, which promoted the degradation of pollutants.

Through theoretical calculations, Yang et al. [144] found that the stable adsorption of low-molecular-weight organic acids (LOAs) on TiO_2_ and the orbital overlap between the highest occupied molecular orbital (HOMO) of LOAs and the lowest unoccupied molecular orbital (LUMO) of TiO_2_ were the key factors affecting the photocatalytic reduction process of Cr (VI). Further analysis revealed that the orbital configuration between LOAs and TiO_2_ facilitates the formation of covalent bonds between them, enabling direct electron transfer from LOAs to the TiO_2_ surface, thereby effectively promoting the reduction reaction of Cr (VI).

#### 4.2.3. Electrostatic Force

Since the carboxyl and phenolic hydroxyl groups of DOM can ionize, DOM generally carries a negative charge in natural environments [140], whereas photocatalysts such as TiO_2_ may carry either a positive or a negative charge in the environment [13]. As the concentration of DOM increases, the interaction between DOM and the catalyst becomes more complex. At this point, electrostatic repulsion caused by the electrostatic double layer formed during DOM adsorption on the catalyst surface becomes dominant. This electrostatic repulsion stabilizes the catalyst, enabling it to maintain relatively consistent catalytic performance despite variations in DOM concentration [145].

#### 4.2.4. Hydrophobic Interaction

DOM containing a relatively high proportion of aromatic functional groups can adsorb onto the surface of photocatalysts through hydrophobic interaction [146]. Luo et al. [147] observed that HA and FA could form different hydrophobic–hydrophilic layers on TiO_2_ NPs. Furthermore, HA, with its higher molecular weight and greater abundance of hydrophobic groups, exhibits a stronger steric stabilization effect on the catalytic material. In contrast, DOM derived from pig manure, sludge, and sediments has a lower molecular weight and aromaticity, resulting in a limited impact on the catalytic material [148].

## 5. Effect of DOM on Active Species

**Figure 4:**
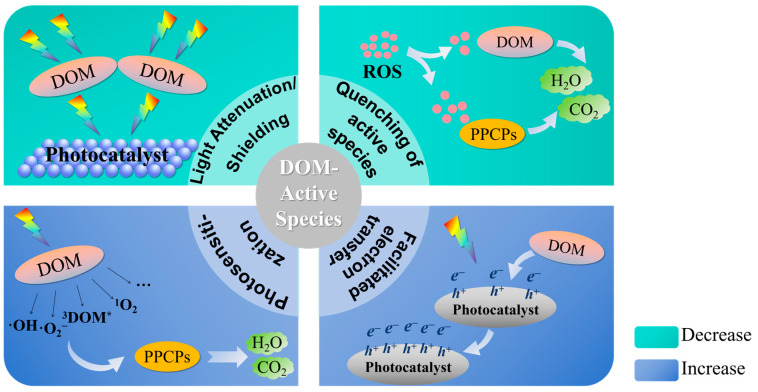
Effect of DOM on active species.

Reactive oxygen species (ROS) are a class of chemically active oxygen-containing molecules or free radicals. ROS mainly includes superoxide anions (·O_2_^−^), hydrogen peroxide (H_2_O_2_), hydroxyl radicals (·OH), and singlet oxygen (^1^O_2_). These substances are highly reactive due to the presence of unpaired electrons and can attack pollutant molecules. The addition of DOM can affect the generation of ROS in the photocatalytic system. However, the photosensitization effect of DOM or its combination with the photocatalyst can also generate ROS, thereby promoting the degradation of pollutants in water [149]. Figure 4 shows the way DOM affects active species.

### 5.1. Generation of Active Species

**Figure 5:**
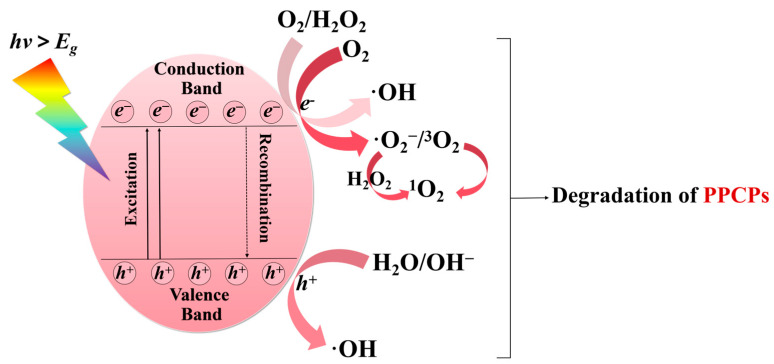
Schematic diagram of the photocatalytic degradation of PPCPs.

According to the energy band theory, semiconductor photocatalysts possess a unique electronic band structure, comprising a lower energy valence band (VB) filled with electrons and a higher energy empty conduction band (CB) [150]. The schematic diagram of the photocatalytic degradation of pollutants is shown in Figure 5. When the photon energy (*hν*) is greater than or equal to the bandgap energy (Eg), the electrons (e^−^) in the VB will be excited into the CB, leaving holes (h^+^) in the VB. The photogenerated holes possess strong oxidizing power and can oxidize the substances adsorbed on the catalyst surface. In an aqueous solution, H_2_O or OH^−^ captures the holes to generate ·OH, while the electrons react with oxygen to form ·O_2_^−^. The ·O_2_^−^ radicals are unstable and highly active, and they will be converted into ·OH through a series of reactions [151]. The generation of singlet oxygen can occur in various ways. For example, h^+^ or e^−^ can directly transfer energy to dissolved oxygen, causing it to transition from the ground state (triplet state, ^3^O_2_) to the excited state (singlet state, ^1^O_2_). Meanwhile, ·O_2_^−^ generates H_2_O_2_ through protonation or disproportionation reactions, and H_2_O_2_ can decompose into ^1^O_2_.

#### 5.1.1. Light Attenuation/Shielding of DOM

DOM competes with pollutants for photoreactive species, thereby interfering with the degradation of organic pollutants [152]. Functional groups in DOM (e.g., carbonyl, carboxyl, and aromatic rings) can absorb light energy. Moreover, as the DOM concentration increases, a light shielding effect occurs, resulting in light attenuation, which leads to a decrease in the number of photons available for the degradation process of organic pollutants. Wang et al. [34] demonstrated that increasing HA concentrations lead to intermediate accumulation on ZnO surfaces, blocking photons from reaching the catalyst surface, thus inhibiting the degradation of TC. Peng et al. [153] indicated that DOM adsorption onto the surface of TiO_2_ particles forms a DOM layer that covers the surface, reducing the utilization efficiency of luminous energy.

#### 5.1.2. DOM Facilitated Electron Transfer

The interaction between DOM and the catalyst influences the generation of active species in the system, with the types and quantities of functional groups in DOM being the main factors. Generally, DOM containing more phenolic hydroxyl groups and methoxy groups is more likely to bind to the active sites on the photocatalysts’ surfaces to form a stable complex. This promotes the separation of photogenerated electrons and holes, thereby enhancing the redox capacity of the photocatalyst.

The electrons generated by DOM can be captured by the catalyst, thus facilitating the electron transfer of the photocatalytic semiconductor [154]. Wu et al. [155] grafted citric acid (CA) onto CeO_2_, which initiated electron transfer, accelerated the regeneration of Ce^3+^ sites as electron donors, and significantly enhanced the photocatalytic performance through the ligand-to-metal charge transfer. Liu et al. [156] showed that HA contains a relatively large number of fluorophores. The time-resolved fluorescence results revealed that the interaction between DOM and TiO_2_ facilitates electron or energy transfer from ^1^DOM* to TiO_2_. Li et al. [84] further confirmed that the generation of ·O_2_^−^ in the TiO_2_/HA mixture was due to the reduction of O_2_ by the electrons transferred from the photodissociated HA to TiO_2_. At the same time, DOM increased the generation of ^1^O_2_ on TiO_2_, CuO, CeO_2_, and SiO_2_, with FA exhibiting a greater promoting effect than HA. Sun et al. [157] found that low-molecular-weight organic acids (LMWOAs) prevented the recombination of photogenerated electron–hole pairs, creating strong reducing sites ·CO_2_^−^, thereby enhancing the photocatalytic degradation effect of nitrazole. Similarly, Feng et al. [158]’s research indicated that low concentrations (2–20 mg·L^−1^) of FA could act as an electron shuttle, thereby promoting the degradation of monochlorobenzene by Fe-Mn bimetallic sulfide. This is because the metal ions on the surface of the catalyst can form complexes with DOM. When DOM rich in quinone functional groups complexes with and adsorbs metal ions on the surface of the catalyst [159], it can exhibit the effect of promoting electron transfer, thereby accelerating the degradation of pollutants.

#### 5.1.3. Photosensitization of DOM

When DOM exists independently in a system, it exhibits photosensitive properties and can participate in complex photochemical reactions [160]. Studies have shown that chromophoric DOM (CDOM) is the main light absorber and photosensitizer in aquatic environments at wavelengths below 500 nm. Under sunlight irradiation, CDOM can generate photoproduction reaction intermediates (PPRIs), including ·OH, ^1^O_2_, ·CO_3_^−^, e_aq_^−^, ·O_2_^−^, H_2_O_2_, and ^3^DOM*, all of which contribute to the degradation of PPCPs. In particular, aromatic components in DOM, such as lignin, are relatively sensitive to solar radiation [161].

Upon light absorption, DOM is photochemically excited from the electronic ground state (S_0_) to the excited singlet state (^1^DOM*). It releases energy and returns to the ground state through fluorescence emission and non-radiative transitions or evolves into the triplet state via intersystem crossing (ISC) under favorable conditions, thus forming ^3^DOM* [141]. ^3^DOM* typically participates in pollutant degradation through electron or hydrogen atom capture or energy transfer, effectively degrading pollutants such as phenols, phenylurea herbicides, and sulfonamide antibiotics. Additionally, the generated ^3^DOM* can activate oxygen through energy transfer to produce ^1^O_2_ [162]. The reaction with ^3^DOM* represents a primary photodegradation pathway for certain PPCPs, such as sulfonamides, β-blockers, and macrolides [163]. Liu et al. [164] studied Suwannee River natural organic matter (NOM) and found that terrestrial humus in the low-to-medium molecular weight (L-MW DOM) fraction, characterized by higher humification and fluorophore content, generates more ^3^DOM* and ^1^O_2_. These two reactive intermediates (RIs) play a significant role in the photodegradation of quinolones. Furthermore, ^3^DOM* is also crucial in the photochemical transformation of amoxicillin in natural water [165].

Under visible light irradiation, low concentrations of FA (<10 mg·L^−1^) generate ^1^O_2_ and ^3^FA*, which strongly promote the degradation of DCF in the vis-RGO/TiO_2_/persulfate system [52]. Cheng et al. [166] showed that, compared to PPCPs containing electron-withdrawing groups (e.g., -NOR, -COOR, -OCR), PPCPs with multiple electron-donating groups (e.g., -OH, -NH_2_, -OR) exhibit higher reactivity with photochemical reaction intermediates such as ^3^DOM* and ^1^O_2_.

### 5.2. Quenching of Active Species

The active groups contained in DOM, such as aromatic amines, alkene compounds, and aromatic alcohols, are capable of interacting with ROS, such as ^1^O_2_. These chemical constituents may undergo redox reactions with ROS through electron transfer pathways, subsequently mediating ROS scavenging mechanisms [167].

Qiao et al. [39] demonstrated that HA reacted with photogenerated holes, which inhibited the generation of ·OH. Generally speaking, DOM can inhibit the generation of ·OH by photocatalysts such as TiO_2_, ZnO, and Fe_2_O_3_. Comparative analyses reveal that FA exhibits stronger inhibitory effects than HA [84]. This is attributable to the second-order rate constant of the reaction between ·OH and FA (2.7 × 10^4^ s^1^(mg of C·L^−1^)^−1^) is approximately 1.7 times higher than that of the reaction between ·OH and HA (1.9 × 10^4^ s^−1^(mg of C·L^−1^)^−1^) [168]. Therefore, the presence of DOM can reduce the utilization of luminous energy in the photocatalytic system and decrease the generation of ROS and h^+^. Generally, the light shielding effect of DOM depends on the wavelength of light, and this effect is more obvious in the ultraviolet range with shorter wavelengths [33].

## 6. Conclusions and Future Perspectives

At present, research efforts on the photocatalytic oxidation of PPCPs primarily focus on the modification or development of photocatalysts and the influence of environmental factors and water matrices, including anions, cations, DOM, and so on. This review synthesized the influence of DOM on the efficiency of PPCPs’ photocatalytic oxidation and reviewed its impact on PPCP migration and transformation, the surface and interface structure of photocatalytic materials, and the active species in the reaction system. The main conclusions are as follows: DOM affects PPCPs migration and transformation through adsorption, complexation, and other effects. DOM affects the adsorption of PPCPs on the photocatalysts’ surfaces through competitive adsorption or by providing more adsorption sites. DOM affects the apparent structure of photocatalysts through ligand exchange, intermolecular forces, electrostatic forces, and hydrophobic interactions. DOM inhibits the generation of active species through light attenuation/shielding and also promotes the generation of active species through photosensitization and the promotion of electron transfer.

The influence of DOM on the photocatalytic degradation of PPCPs is relatively complex, and there are some drawbacks in the current research:

1. Most of the existing studies focus on the influence of DOM on the degradation efficiency of PPCPs, but there are few studies on how DOM regulates key processes such as the generation pathway of reactive oxygen species and the separation efficiency of photogenerated electron–hole pairs.
2. The types of DOM are complex, such as HA, polysaccharides, and proteins, and the influence of different components on photocatalysis varies significantly. However, most of the current studies use a single standard DOM, such as HA, for simulation experiments, which has a large deviation from the DOM composition in the actual environment, resulting in limited universality of the conclusions.
3. DOM participating in the photocatalytic process may generate more toxic intermediate products, such as halogenated by-products, but most of the related studies focus on the short-term degradation effect, and there is insufficient evaluation of the stability of the catalyst and the ecological risk of degradation products during long-term operation.

Future research can be carried out in the following aspects:

1. Future research should combine in situ characterization techniques, such as in situ fluorescence spectroscopy, electron paramagnetic resonance, and theoretical calculations, such as density functional theory, and other methods to systematically explore the influence of DOM on the generation pathway of reactive oxygen species and the mechanism of DOM in the separation of photogenerated electron–hole pairs.
2. Using high-throughput sequencing, mass spectrometry analysis, and other technical means to accurately quantify different components in DOM and evaluate their respective influences on the photocatalytic process. By comparing the results of simulation experiments with a single standard DOM, such as HA, and the actual DOM composition, improve the universality and accuracy of the results.
3. By extending the experimental cycle and using various ecological toxicity testing methods, comprehensively evaluate the potential impact of DOM participating in the photocatalytic process on the aquatic ecosystem.

## Figures and Tables

**Table 1:** Effect of DOM on the photocatalytic oxidation of PPCPs.

Photocatalysts	Types of Light Sources	Catalyst Dosage (g·L^−1^)	PPCPs	DOM and Its Concentration	Level of Influence	Reference
TiO_2_	UV	1.0	BUP	HA, 10 mg·L^−1^	−, 80%	[31]
TiO_2_ (anatase)	UV	0.2	4-CP	HA, 1–30 mg·L^−1^	−	[32]
TiO_2_ (rutile)				HA, <20 mg·L^−1^	+	
				HA, 30 mg·L^−1^	−	
TiO_2_ TNAs	UV	/	MTL	NOM, 15 mg·L^−1^	−, 48.16%	[33]
ZnO	vis	0.8	TC	HA, 5 mg·L^−1^	−, 19%	[34]
ZnO NPs	vis	0.01	MB	HA, 10 mg·L^−1^	−, 79.4%	[35]
ZnO nanowires	vis	0.02	CPX	SRNOM, 10 mg·L^−1^	−, 59.1%	[36]
Mn-WO_3_	LED	2.2	DCF	HA	−	[37]
Oxygen-doped porous g-C_3_N_4_	vis	1.0	CBZ	HA, 20 mM	+	[38]
g-C_3_N_4_ nanosheets	vis	1.0	PHE	HA, 10 mg·L^−1^	−, 18%	[39]
GO	vis	0.1	APAP	FA, 20 mg·L^−1^	−, 84.8%	[40]
CdS@BC	vis	1.0	THM	HA	−	[41]
SrBiOI	vis	0.4	IDM	DOM, 10 mg·L^−1^	−, 30%	[42]
MI-BiOCl	vis	0.4	VEN	HA, 20 mg·L^−1^	o	[43]
Bi-TNB	vis	0.5	NPX	HA, 5 mg·L^−1^	+, 2 times	[44]
				FA, 10 mg·L^−1^	−	
SnO_2_@ZnS	vis	/	MTL	DOM	−, 52%	[45]
BiOBr/Fe_3_O_4_	vis	0.5	NOR	HA, 10 mM	−, 44.49%	[46]
BiOBr/Ti_3_C_2_	vis	2.4	FQNs	HA	o	[47]
Bi_2_MoO_6_/(BiO)_2_CO_3_	vis	0.5	APAP	NOM, 10 mg·L^−1^	−, 21%	[48]
Er^3+^-CdS/MoS_2_	vis	0.125	17β-E2	HA	+	[49]
WO_3_ Fibers/g-C_3_N_4_	vis	0.1	CTD	HA, 20 mg·L^−1^	−, 12.9%	[50]
g-C_3_N_4_/Ag_3_PO_4_	vis	0.5	OFX	HA	−	[51]
rGO/TiO_2_	Blue light	0.3	DCF	FA, ≤10 mg·L^−1^	+	[52]
				FA > 10 mg·L^−1^	−	
Bi_2_Fe_4_O_9_/rGO	vis	0.2	TC	HA	−	[53]
MWCNTs/Bi_4_O_5_I_2_ nanosheets	vis	0.2	TC	HA, 8 mg·L^−1^	−, 5%	[54]
CoFe_2_O_4_-rGO	UVA- LED	0.4	BPA	HA, 10 mg·L^−1^	−	[55]
Ag_2_WO_4_/PCN	vis	0.2	IDM	DOM, 10 mg·L^−1^	−, 26%	[56]
PO4^3−^-Bi_2_WO_6_/PI	vis	1.0	TC	HA	−	[57]
Benzene-ring doped CN/Phosphorus-doped CN	Blue light (LED)	0.2	SSZ	DOM, 10 mg·L^−1^	−, 74.8%	[58]
WO_3/_ZnIn_2_S_4_-3	LED	1.6	TBBPA	HA, 1 mmol·L^−1^	−, 53.9%	[59]
C_CPD_-g-C_3_N_4_	vis	1.0	MBP	SRHA, 50 mg·L^−1^	−, 20%	[60]
CQD-SnNb_2_O_6_/BiOCl	vis	0.5	BC	HA, 10 mg·L^−1^	−, 18%	[61]
Cd_0.5_Zn_0.5_S/BiOCl	vis	0.2	NOR	HA, 20 mg·L^−1^	−, 35%	[62]
IS-Ni_2_P/CdS/CN	vis	0.1	TC	HA	+	[63]
V_W+Br_-BiOBr/Bi_2_WO_6_	vis	0.3	NOR	HA, 10 mg·L^−1^	−, 21.9%	[64]
BMOF-Ti/Zr_6%_	UV	/	BP-2	DOM, 20 μg·L^−1^	−	[65]
Zr-MOFs	vis	/	STZ	HA	−	[66]
π-COF	vis	0.2	TC	HA	−	[67]
ZnFe_2_O_4_-seed@TpTt-COF	vis	0.1	BPA	HA	+	[68]
MIL-88B(Fe)/ZnTi-LDH high-low junction	vis	0.2	TC	HA	+	[69]

Promotion (+); inhibitory (−); no noteworthy influence (o).

## Data Availability

Not applicable.

## Abbreviations

The following abbreviations are used in this manuscript:

APAP	Acetaminophen (paracetamol)
BC	Benzocaine
BDOM	Biochar dissolved organic matter
Bi-TNB	Bismuth titanate nanobulk
BP-2	2,2′,4,4′-Tetrehydroxybenzophenone
BPA	Bisphenol A
BUP	Bupropion
CA	Citric acid
CBZ	Carbamazepine
CDOM	Chromophoric dissolved organic matter
CHA	Coal humic acid
COFs	Covalent organic frameworks
CPX	Cephalexin
CTD	Clothianidin
DCF	Diclofenac
DEET	N,N-Diethyl-3-methyl benzoyl amide
DOC	Dissolved organic carbon
DOM	Dissolved organic matter
FA	Fulvic acid
FQNs	Fluoroquinolones
GO	Graphene oxide
HA	Humic acid
IDM	Indomethacin
LOAs	Low-molecular-weight organic acids
MWCNTs	Multi-walled carbon nanotubes
NOM	Natural organic matter
NOR	Norfloxacin
NPX	Naproxen
NPs	Nanoparticles
OFX	Ofloxacin
PHE	Phenanthrene
PPCPs	Pharmaceuticals and personal care products
PPRIs	Photoproduction reaction intermediates
rGO	Reduced graphene oxide
RIs	Reactive intermediates
ROS	Reactive oxidative species
SHA	Soil humic acid
SMX	Sulfamethoxazole
SSZ	Sulfasalazine
STZ	Sulfathiazole
TBBPA	Tetrabromobisphenol A
TC	Tetracycline
TCS	Triclosan
THM	Trimethoprim
UV	Ultraviolet
VEN	Venlafaxine
vis	Visible light
17β-E2	17β-Estradiol
4-CP	4-chlorophenol
π-π EDA	π-π electron donor–acceptor
